# A Multidimensional Digital Health Platform to Support Intrinsic Capacity and Healthy Aging: Pilot Feasibility Study

**DOI:** 10.2196/79862

**Published:** 2026-07-28

**Authors:** Marcin Kolakowski, Andrea Lupica, Seif Ben Bader, Jaouhar Ayadi, Luca Gilardi, Angelo Consoli, Irina Georgiana Mocanu, Oana Cramariuc, Lionello Ferrazzini, Eva Reithner, Magdalena Velciu, Barbara Borgogni, Sofia Rivaira, Luca Antognoli, Sara Leonzi, Elisa Felici, Margherita Rampioni, Giacomo Cucchieri, Vera Stara

**Affiliations:** 1 Institute of Radioelectronics and Multimedia Technology Warsaw University of Technology Warsaw Poland; 2 ECLEXYS Sagl Riva San Vitale Switzerland; 3 OCTILIUM Sagl Chiasso Switzerland; 4 Centrul IT Pentru Stiinta si Tehnologie (CITST) Bucharest Romania; 5 EURAG Österreich Wien Austria; 6 Ana Aslan International Foundation Bucharest Romania; 7 Medea S.r.l. Florence Italy; 8 IRCCS INRCA Istituto Nazionale di Riposo e Cura per Anziani Ancona Italy

**Keywords:** digital health, healthy aging, intrinsic capacity, older adults, usability, user experience, remote monitoring, integrated care platform, artificial intelligence, multidimensional health assessment

## Abstract

**Background:**

By 2050, 22% of the global population will be aged 60 years or older, with Europe experiencing rapid aging. This increase in chronic diseases and functional decline demands a shift from fragmented, hospital-centric care to continuous care models. The World Health Organization’s healthy aging framework prioritizes intrinsic capacity (IC)—defined as physical and mental abilities across the locomotion, vitality, sensory, cognition, and psychology domains. Digital health solutions enable remote monitoring but face barriers, such as low digital literacy and usability challenges among older adults.

**Objective:**

This study evaluates the impact of a multidimensional, artificial intelligence–driven digital health platform (CAREUP) on the dynamic monitoring and personalized enhancement of IC in community-dwelling older adults through the integration of real-time physiological, cognitive, and behavioral data streams.

**Methods:**

This pilot feasibility study was conducted from September to December 2024 in Italy, Romania, and Austria. It involved 66 older adults as primary users and 17 caregivers as secondary users. The CAREUP system included Android apps (Careplan and Positive Health), smart devices, and a dashboard. Users followed a 2-month plan. We assessed usability (System Usability Scale [SUS]), user experience (User Experience Questionnaire short version [UEQ-S]), and acceptance (qualitative) at 3 time points (T0, T1, and T2), along with health and quality-of-life measures.

**Results:**

Of the 66 enrolled participants, 61 primary users (37 women and 24 men; mean age 73.9 years) completed the study. The overall SUS score was 68, indicating acceptable but improvable usability, with significant variation across countries. The UEQ-S score of 1.45 indicated a good overall user experience, with high Hedonic Quality (1.66) suggesting strong emotional appeal and Pragmatic Quality (1.23) reflecting above-average usability. Most participants (49/61, 80%) rated the platform as interesting, helpful, and efficient, and 48 out of 61 (79%) were willing to recommend it to friends. Secondary outcomes demonstrated stable physical and mental health and maintained independence in daily activities. Technical connectivity issues and device complexity emerged as the primary barriers to adoption.

**Conclusions:**

CAREUP demonstrated feasibility for IC monitoring, with acceptable usability and a positive user experience. However, technical challenges and cross-country variations indicate a need for adaptations to address cultural differences and varying levels of digital literacy. Prioritizing reliability, simplifying the system, and providing user support will be essential to enhance broader adoption.

## Introduction

### Background

By 2050, nearly 22% of the global population will be aged 60 years or older, with Europe experiencing a particularly rapid demographic shift in which those aged over 65 years will outnumber children aged under 15 years [1,2]. This aging trend is accompanied by a rise in chronic diseases, as aging is a primary risk factor for many health conditions [3,4]. Age-related chronic diseases often lead to functional decline and social isolation, making it difficult for older adults to maintain independence and quality of life.

To shift away from a disease-centered model of aging, the World Health Organization introduced the concept of healthy aging, which focuses on preserving functional abilities through the optimization of intrinsic capacity (IC) alongside environmental support. IC encompasses the core physical and mental abilities individuals have at different stages of life, which are influenced by environmental factors and naturally decline with age. It includes 5 key domains: locomotion, vitality, sensory function, cognition, and psychological health [5-7].

Preventing chronic diseases and improving the lives of older adults require care models that focus on maintaining IC. The World Health Organization’s Integrated Care for Older People framework recommends an integrative, long-term, multidisciplinary approach centered on person-focused care [8]. However, these complex care needs place significant strain on health care and social systems that are already facing resource shortages, compounded by a limited supply of formal and informal caregivers [9-11].

Digital technologies present promising solutions to support this challenging landscape. Remote monitoring and digital health platforms can streamline and integrate the management of chronic conditions, enhancing older adults’ independence while reducing health care costs. Collectively referred to as digital health solutions, these technologies require users to have varying levels of health and digital literacy, which must be considered to ensure accessibility and adoption, especially among older adults with frailty and chronic disability [12-16]. Given the wide variability in digital skills among seniors and their preference for personal contact with health care providers, digital tools must be tailored to be simple, intuitive, and user-friendly—for example, by employing large fonts, minimal button use, and straightforward interactions [17-19].

### Knowledge Gap

Many digital platforms targeting healthy aging have been developed in both commercial and research settings, as summarized in Table 1. Most health monitoring platforms collect data using sensors, such as blood pressure monitors, thermometers, oximeters, smartwatches, and activity trackers, as well as digital questionnaires assessing mental well-being and social engagement. These data are typically uploaded via a gateway device, such as a tablet or smartphone, and processed in the cloud. The outcomes are usually accessible through web- or mobile-based interfaces.

**Table 1:** Exemplary health monitoring platforms.

Platform name	Target	Data sources	Interface	Additional features	Trials	Usability
Comarch HomeHealth 2.0 [20]	Patients and medical professionals	Sensors and questionnaires	Web and mobile	Alerts of parameters outside of predefined scope and virtual visits	Not reported	Not reported
RO-SmartAgeing [21,22]	Patients and medical professionals	Sensors	Web and mobile	Alarms for unusual parameter values	Laboratory tested	High (laboratory tested)
InnoWell [23]	Patients and medical professionals	Sensors, medical records, and questionnaires	Web	No	Not yet reported; protocol available	Low
My AHA [24]	Patients and medical professionals	Sensors and other platforms (acts as middleware)	Web and mobile	Personalized care plan and fall and dementia risk	20 adults (alpha wave)	Not reported directly; platform revised after alpha wave
GER-e-TEC [25,26]	Older adults	Sensors and questionnaires	Web	Alerts of warning signs leading to hospitalization	36 adults monitored for 2 months; specificity could be higher	Not reported
FRAIL [27]	Patients with prefrailty and frailty	Sensors (including custom vest)	Web	Gamification system	40 adults recruited to test the devices	Medium usability of the custom sensors
CAREPATH [28,29]	Older adults	Sensors and questionnaires	Web and mobile	Personalized care plan and physical exercises plan	11 project team members	Good to excellent according to the project team members
HOPES [30,31]	Patients and medical professionals	Sensors and smartphone	Web and mobile	Anomaly detection	100 patients with schizophrenia	Not reported
Vivify +Home [32]	Patients and medical professionals	Sensors and smartphone	Web and mobile	Health tips and virtual visits	Not reported	Not reported
NESTORE [33]	Older adults	Sensors, questionnaires, and location data	Web and mobile	Personalized care plan, social platform, and chatbot	10 older adults	Reported issues with software usability
CAREUP [34]	Older adults	Sensors, questionnaires, and games	Web and mobile	Personalized care plan, intrinsic capacity monitoring, intrinsic capacity prediction, and games	66 older adults and 17 caregivers in 2 months	See the “Usability” section

The platforms differ primarily in how they process data and the services they offer. Medical system–connected platforms often alert caregivers or clinicians to abnormal physiological readings and may provide online consultation features. Home-use platforms tend to focus on personalized care plans that adapt based on the user’s current status. More advanced solutions incorporate predictive analytics to estimate the risk of future health problems or declines in capacity.

As maintaining user engagement is crucial for platform success, features such as social networking and gamification are commonly added to enhance adherence. However, usability remains a pervasive and critical challenge. Many platforms incorporate wearables, mobile apps, and surveys to cover all IC domains, but often neglect the linguistic, educational, and social diversity of older adults. This oversight results in low user acceptance and limited engagement [23,35-40]. Reported challenges include difficulties with navigation, lack of personalized experiences, software bugs, and hardware integration issues [27,33]. These barriers are frequently observed in newly developed platforms within research projects.

Caring for an aging population within the healthy aging framework requires supporting residual IC and compensating for its decline. Technology designed specifically for older adults may facilitate integrated and continuous care, thereby addressing physical and psychosocial needs more effectively.

### Study Objectives

The CAREUP project introduced an innovative digital platform for the remote monitoring of older individuals’ IC to promote inclusive health. It integrates real-time physiological, cognitive, and behavioral data, aiming to slow IC decline and support caregiver-assisted care. This paper reports on a pilot feasibility study detailing the study design and user interactions with the CAREUP platform across Italy, Romania, and Austria. The results focus on usability, acceptance, and the platform’s impact on quality of life, cognition, and physical activity. In line with current reporting standards for digital health implementations, this work was structured according to the iCHECK‑DH (Guidelines and Checklist for the Reporting on Digital Health Implementations) to enhance completeness, transparency, and reproducibility of the implementation description. The completed iCHECK‑DH checklist is provided as Multimedia Appendix 1.

## Methods

### Study Design

The pilot feasibility study was one of the main objectives of the CAREUP project, aimed at evaluating the platform in a real-life environment and collecting feedback from actual users. A mixed methods design was used to collect data from primary (older adults) and secondary users (formal or informal caregivers). End users (primary and secondary) were enrolled by project partners in 3 European countries (Austria, Italy, and Romania) from September 2024 to December 2024.

### Ethical Considerations

The research protocol was approved by the Ethics Committee of IRCCS INRCA (Istituto Nazionale di Ricovero e Cura per Anziani) on March 30, 2023 (approval number D/GEN 91).

### CAREUP Platform Technical Description

**Figure 1:**
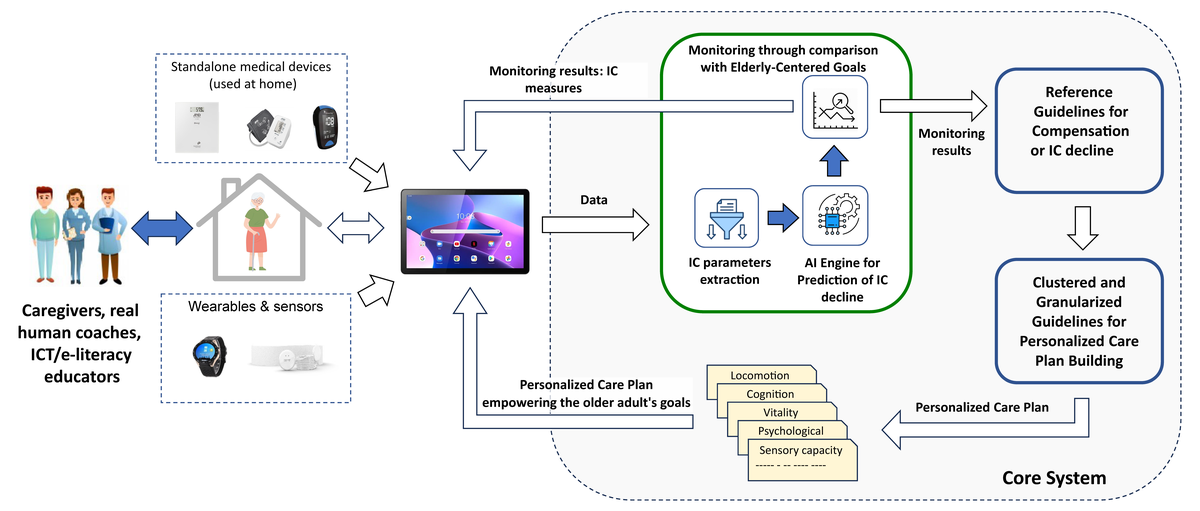
The CAREUP platform concept. AI: artificial intelligence; IC: intrinsic capacity; ICT: information and communications technology.

The CAREUP platform integrates Android apps installed on a tablet (Careplan and Positive Health [PH]), smart devices, and an online dashboard (CAREUP’s Portal Web Interface). The CAREUP platform concept is presented in Figure 1, and a brief description of the platform components is provided in Table 2. The technical aspects of the platform and the implemented algorithms are described in more detail elsewhere [34].

**Table 2:** CAREUP platform: components and functions.

Component type and the respective component		Function
Android tablet apps		
	Careplan app	Health parameters scan and report Access to cognitive games apps
	Positive Health app	Health questionnaires and locomotion test
**Devices**		
	Smartwatch (Starmax WS50)	Step number acquisition
	Blood pressure monitor (iHealth track)	Blood pressure measurement
	Weight scale (iHealth weight scale)	Weight measurements
	Oximeter (iHealth PO3M)	Saturation measurement
	Glucometer (iHealth glucometer)	Blood glucose measurement
	Handgrip strength meter (GRIPX Digital Hand Dynamometer Grip Strength Measurement Meter)	Grip strength measurement
**Online dashboard**		
	CAREUP’s Portal Web Interface	Monitoring of health parameters and intrinsic capacity values

The platform operation follows a common pattern in which primary users, using the platform devices, collect multimodal health data, such as the number of steps taken per day, blood pressure, oxygen saturation, body weight, and sleep quality. The core platform app is the Careplan app, which acts as the entry point to all platform apps. Its main purpose is to collect data from the medical devices. For supported Bluetooth-enabled devices, data are transferred and uploaded automatically using standard, secure protocols; for others, results can be entered manually. The Careplan app presents users with a personalized care plan, including tips for maintaining their IC at the required levels. Users can also access the PH app and the game section through provided links, featuring cognitive games selectable from both commercial and CAREUP-specific options.

The PH app is designed to collect health data that cannot be captured with smart devices. It includes digital health questionnaires to gather information on aspects such as well-being, emotions, and social activities, as well as 2 locomotion tests to assess physical performance. The PH app questionnaires are brief and consist of 3-4 closed-ended questions selected from a pool of 89 items derived from standardized and ad hoc reference questionnaires (Table 3). To reduce respondent burden, the PH app questionnaires do not address family situation, cognition, religion, or instrumental activities of daily living. A questionnaire for a given day is generated according to a predefined 4-week cycle, during which each question is asked at least once. Questions from specific reference questionnaires are distributed throughout the cycle to avoid monotony and maintain user engagement. If a user forgets to complete the PH app on a given day, the prepared questions are presented the following day. The questionnaire generation cycle is described in detail in Multimedia Appendix 2.

**Table 3:** Reference list of health questionnaires.

Standardized questionnaires		Assessment area
EQ-5D-5L [41]		Quality of life
15-item Geriatric Depression Scale [42]		Depression level
Health Status 12-Item Short Form version 2 [43]		Quality of life
Instrumental Activities of Daily Living scale [44]		Overall well-being
Loneliness Scale version 3 [45]		Perceived social isolation
Lubben Social Network Scale—Revised [46]		Social isolation
Mini Nutritional Assessment [47]		Overall nutritional status
Montreal Cognitive Assessment [48]		Mild cognitive impairment
Patient Health Questionnaire [49]		Depression level
Sensory domain (ad hoc questionnaire)		Sensory system
Short Physical Performance Battery [50]		Functional mobility in older adults
Sleep Quality Scale [51]		Sleep quality
Spiritual Well-Being scale [52]		Perceived spiritual quality of life
User-Centered Goals (ad hoc questionnaire)		User’s goals/needs
**Locomotion test**		
	30-second chair stand test [53]	Leg strength and endurance
	Balance test [54]	Balance

The app’s locomotion tests implemented selected components of the Short Physical Performance Battery, specifically the chair stand and balance tests. The PH app also provides users with appropriate instructions to enable them to perform the tests at home.

Results from device measurements and from the PH app’s questionnaires and tests are uploaded to the platform via a Representational State Transfer (REST) application programming interface using the secure HTTPS protocol. In addition, any application programming interface operation requires a valid token to be provided with the request. Tokens are generated only for logged-in users and have an expiration date. After a prolonged break of a few days in platform use, the user must log in again.

The results are stored in a secure database in the CAREUP cloud. For additional data security, they are pseudonymized, and they cannot be directly linked to users’ personal information without access to additional data. The collected results are used for weekly assessments of primary users’ health status and IC. They also serve as the basis for preparing personalized care plans and IC prognoses.

Summaries and graphs of the analysis results are automatically generated and presented in the CAREUP Portal Web Interface, the online dashboard of the CAREUP platform. This browser-based interface can be accessed by both primary and secondary users using personal credentials to review health status and monitor changes in IC among older adults. In addition, the Web Portal provides access to results from medical devices and outcomes of game sessions completed by the primary user.

### Outcomes Measures

Data collection was conducted at 3 time points: the baseline evaluation (T0), the intermediate evaluation (T1), and the final evaluation (T2).

The primary outcomes, assessed at T1 and T2, aimed to evaluate the system’s usability, user experience, and acceptance. Perceived usability of the platform (mobile apps, devices, and online dashboard) was assessed using the System Usability Scale (SUS) [55]; user experience was assessed using the User Experience Questionnaire short version (UEQ-S) [56]; and acceptance was evaluated using an ad hoc qualitative questionnaire consisting of open-ended questions.

The SUS score indicates excellent usability if it is over 80.3 (grade A), good usability from 68 to 80.3 (grade B), and poor or unacceptable usability if it is less than 51 (grade F). The UEQ-S focuses on pragmatic quality (usability-related aspects such as efficiency, clarity, and task support) and hedonic quality (emotional and aesthetic appeal, excitement, and innovation). Its score ranges from –3 to +3 and represents a very bad (–3.00 to –0.81), neutral (–0.80 to 0.80), good (0.81-1.40), excellent (1.41-2.00), or outstanding (2.01-3.00) experience. Acceptance refers to the degree to which users are willing to use a form of technology and was assessed using ad hoc open-ended questions.

The secondary outcomes included various health variables evaluated at T0, T1, and T2. Physical health was assessed using the 12-Item Short Form version 2 (SF-12 v2) survey [43] and the Instrumental Activities of Daily Living (IADL) scale [44]. Perceived loneliness was assessed using the UCLA Loneliness Scale (UCLA version 3 [UCLA v3]) [45], and social interaction was assessed using the Lubben Social Network Scale—Revised (LSNS-R) [46]. Quality of life was assessed using the EQ-5D-5L [41]. Cognitive and mood health were assessed using the Montreal Cognitive Assessment (MOCA) [48] and the 15-item Geriatric Depression Scale (GDS-15) [42], respectively. Spiritual well-being was assessed using the Spiritual Well-Being Scale score [52], and cognitive game experience was assessed using the Game Experience Questionnaire [57]. In addition, care workload among secondary users was assessed as a secondary outcome using ad hoc questions.

The SF-12 [43] provides 2 summary scores: 1 for the physical component summary and 1 for the mental component summary. The UCLA v3 [45] score can range from 20 to 80, with higher scores indicating greater feelings of loneliness and isolation (scores: 20-30, low loneliness; 31-50, moderate loneliness; and 51-80, high loneliness). The LSNS-R [46] focuses on the quality and quantity of social relationships; higher scores indicate a wider and more supportive social network (scores: 12-30, low social support; 31-44, satisfactory network with room for improvement; and 45-60, strong and supportive social network). The GDS-15 [42] score ranges from 0 to 15. Values up to 5 indicate a normal condition, while scores from 5 to 10 indicate moderate depression and scores from 11 to 15 indicate severe depression. The Spiritual Well-Being Scale [52] total score ranges from 20 to 120, with higher scores indicating greater spiritual well-being. The EQ-5D-5L [41] measures health-related quality of life using 5 items, and the Visual Analogue Scale, with values ranging from 0 to 100, measures the person’s perceived state of health. The MOCA [48] has a maximum score of 30 points and generally indicates the presence of cognitive impairment when the score is 26 or lower. The Game Experience Questionnaire [57] measures the player’s emotional and cognitive status after playing games, and its score follows a 5-point Likert scale ranging from 0 (not at all) to 4 (extremely).

### Participants

#### Overview

During the study, data were collected from both primary and secondary users according to predefined inclusion and exclusion criteria. The primary users were older adults, whereas the secondary users were their formal or informal caregivers.

#### Inclusion Criteria

Primary users were deemed eligible for the pilot if they (1) were aged 65 years or older; (2) lived independently in their homes in rural or urban areas; (3) had the capacity to provide consent and were available to sign the informed consent form; and (4) were willing to participate voluntarily in the pilot.

#### Exclusion Criteria

The exclusion criteria were as follows: (1) failure to meet the inclusion criteria; (2) use of active implantable or nonimplantable medical devices; (3) allergy to nickel components; (4) concomitant participation in other studies; (5) lack of written informed consent; (6) acute or untreated medical problems, such as a history of syncopal episodes, epilepsy, or pharmacologically uncontrolled vertigo; serious dysfunction of the autonomic system; severe behavioral syndromes not compensated by medication; concurrent neurological diseases; or severe systemic diseases with a life expectancy of less than 1 year; (7) myocardial infarction or stroke within the previous 6 months; (8) uncontrolled hypertension; (9) pacemaker or implantable cardioverter-defibrillator; (10) advanced Parkinson disease or other neuromuscular disorders; (11) metastatic cancer or immunosuppressive therapy; and (12) significant visual or hearing impairment. No inclusion or exclusion criteria were applied for secondary users.

#### Recruitment Strategy

Recruitment was multicenter and multinational, involving sites in Italy (n=30), Romania (n=23), and Austria (n=8) from September to December 2024. Participants were recruited through collaborating clinics, local outreach, and direct invitations and were representative of both rural and urban populations. Each site provided local training and support.

#### Sample Size Rationale

As a pilot study, the sample size (n=66) was determined to enroll a sufficient number of participants to capture usability barriers, acceptance issues, and preliminary outcomes across diverse cultural contexts. No formal power calculation was performed, as the primary aims were formative rather than confirmatory [58]. Although the sample size was limited, the study was explicitly designed as an exploratory feasibility investigation to evaluate the usability, acceptance, and preliminary impact of the CAREUP platform.

### Procedure

At T0, the primary and secondary users were asked to sign the informed consent form. Sociodemographic data were then collected from all participants. The initial evaluation of the primary users also included the MOCA [48] assessment to evaluate cognitive status and the User-Centered Goals questionnaire to define personal health goals and ambitions. Each primary user was provided, on a free-loan basis for the entire duration of the study, with all necessary equipment, including a tablet equipped with a SIM card for internet access and a selection of smart medical devices. Through the tablet, users could access the CAREUP platform apps and the online dashboard. Users were asked to select the smart devices they wished to test, and only the selected devices were provided.

Participants received detailed explanations on how to use the system and its role in supporting and monitoring their health and IC. An introductory training phase was also conducted at the start of the pilot at each study site. During this phase, users received a manual in their native language along with recommendations for system use. Participants were instructed to access the tablet apps at least once a week; use the devices at least 2-3 times per week; complete the questionnaires and locomotion test once a week; and play the games as desired. All collected data (measurement values and IC graphs) could be checked daily or weekly by both primary and secondary users through the web portal (online dashboard).

Primary users were invited to use the platform according to these recommendations from T0 to T2, covering the entire pilot period of approximately 2 months. Details of the assessments conducted at T0, T1, and T2 are summarized in Table 4.

**Table 4:** List of tests and measurements for the pilot.

Dimension	Scale	Type of outcome	Type of end user	Timing of data collection
Socio Demo Data	N/A^a^	N/A	Primary and secondary	T0
User-Centered Goals	N/A	N/A	Primary	T0
Usability and User Experience	SUS^b^ and UEQ-S^c^	Primary	Primary and secondary	T1 and T2 (for both)
Acceptance	Qualitative items	Primary	Primary and secondary	T1 and T2 (for both)
Cognitive Status	MOCA^d^	Secondary	Primary user	T0 and T2
Physical Health	SF-12^e^ version 2 and IADL^f^	Secondary	Primary user	T0, T1, and T2
Perceived Loneliness	UCLA version 3	Secondary	Primary user	T0, T1, and T2
Social Interaction	LSNS-R^g^	Secondary	Primary user	T0, T1, and T2
Quality of Life	EQ-5D-5L	Secondary	Primary user	T0, T1, and T2
Mental Health	GDS-15^h^	Secondary	Primary user	T0, T1, and T2
Game Experience Questionnaire, part 3 postgame module	Game Experience Questionnaire	Secondary	Primary user	T1 and T2
Privacy and Stigma	Qualitative items	Secondary	Primary and secondary	T1 and T2 (for both)
Care Workload	Qualitative items	Secondary	Secondary user	T1 and T2
Willingness to Pay	Qualitative online questionnaire	Secondary	Primary and secondary	T2

^a^N/A: not applicable.

^b^SUS: System Usability Scale.

^c^UEQ-S: User Experience Questionnaire short version.

^d^MOCA: Montreal Cognitive Assessment.

^e^SF-12: 12-Item Short Form.

^f^IADL: Instrumental Activities of Daily Living.

^g^LSNS-R: Lubben Social Network Scale—Revised.

^h^GDS-15: 15-item Geriatric Depression Scale.

### Statistical Analysis

The following section describes the outcomes of the pilot study. The pilot study data were uploaded to the Zenodo repository and are publicly available [59]. The dataset includes the results of the T0, T1, and T2 assessments; physiological measurements performed using the medical devices; and responses to the PH app questionnaires. The data are accompanied by anonymized demographic information for the users.

The dataset was curated at the end of the pilot study, and all data were cleaned and encoded using Python (Python Foundation) and the pandas package. The data are stored in standard CSV format. Instructions for dataset use, including a description of the file structure and variable dictionary, are available in the dataset description PDF file. The repository also includes exemplary Python code for dataset loading and visualization.

**Figure 2:**
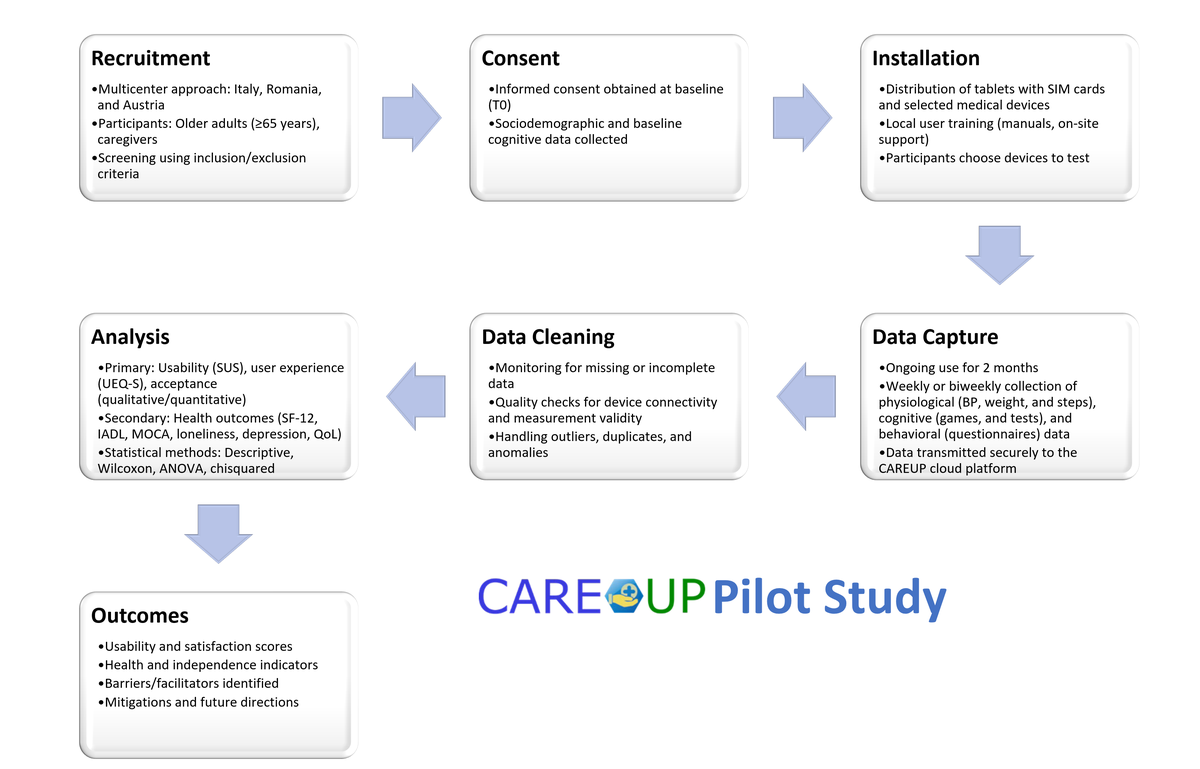
Study flowchart. IADL: Instrumental Activities of Daily Living scale; MOCA: Montreal Cognitive Assessment; QoL: quality of life; SF-12: 12-Item Short Form; SUS: System Usability Scale; UEQ-S: User Experience Questionnaire short version;.

In the following analysis, data are reported using mean values (a measure of central tendency representing the typical value of the dataset), frequencies (indicating how often a particular value or category appears in the dataset), and *P* values (used to determine whether observed differences or associations in the data are statistically significant). Statistical analyses included the Wilcoxon test and ANOVA for continuous variables, whereas the chi-square test was used for categorical variables. These analyses were conducted using Python (version 3.11), leveraging libraries such as SciPy for statistical testing, pandas for data handling, and Seaborn for visualization. A descriptive flowchart of all study stages is presented in Figure 2.

## Results

### User Statistics

A total of 66 older adults participated as primary users: 30 were enrolled in Italy, 25 in Romania, and 11 in Austria. Their caregivers were involved as secondary users (7 in Romania, 5 in Austria, and 5 in Italy).

In Romania, 2 primary users (who enrolled without caregivers) withdrew after the first week of the pilot because they considered the platform too difficult to use and requiring IT knowledge that they did not have. There were also 3 primary user dropouts in Austria: 2 withdrew during the first week after recruitment, and 1 withdrew after the T1 phase. These users did not perceive any benefit from using the system. The pilot was therefore completed by 61 primary users and 17 secondary users. Not every primary user (older adult) was assigned a secondary user (caregiver), as the inclusion criteria for primary users required them to be in good health and not in need of caregiver support for daily activities.

The primary users included 37 women and 24 men, with a mean age of 73.9 years. Most participants were married (n=36), whereas 14 were widowed, 4 divorced, 3 separated, 2 single, and 2 in a full-time relationship. Regarding education level, 27 older adults had a secondary education, 28 had a tertiary education (university or further education), 5 had a primary education, and 1 had no formal education. Most participants (n=54) lived in urban areas, whereas 7 lived in rural areas.

The secondary users included 10 men and 7 women, with a mean age of 47.5 years. All were informal caregivers except for 1 formal caregiver from Italy. Full sociodemographic details are presented in Table 5.

**Figure 3:**
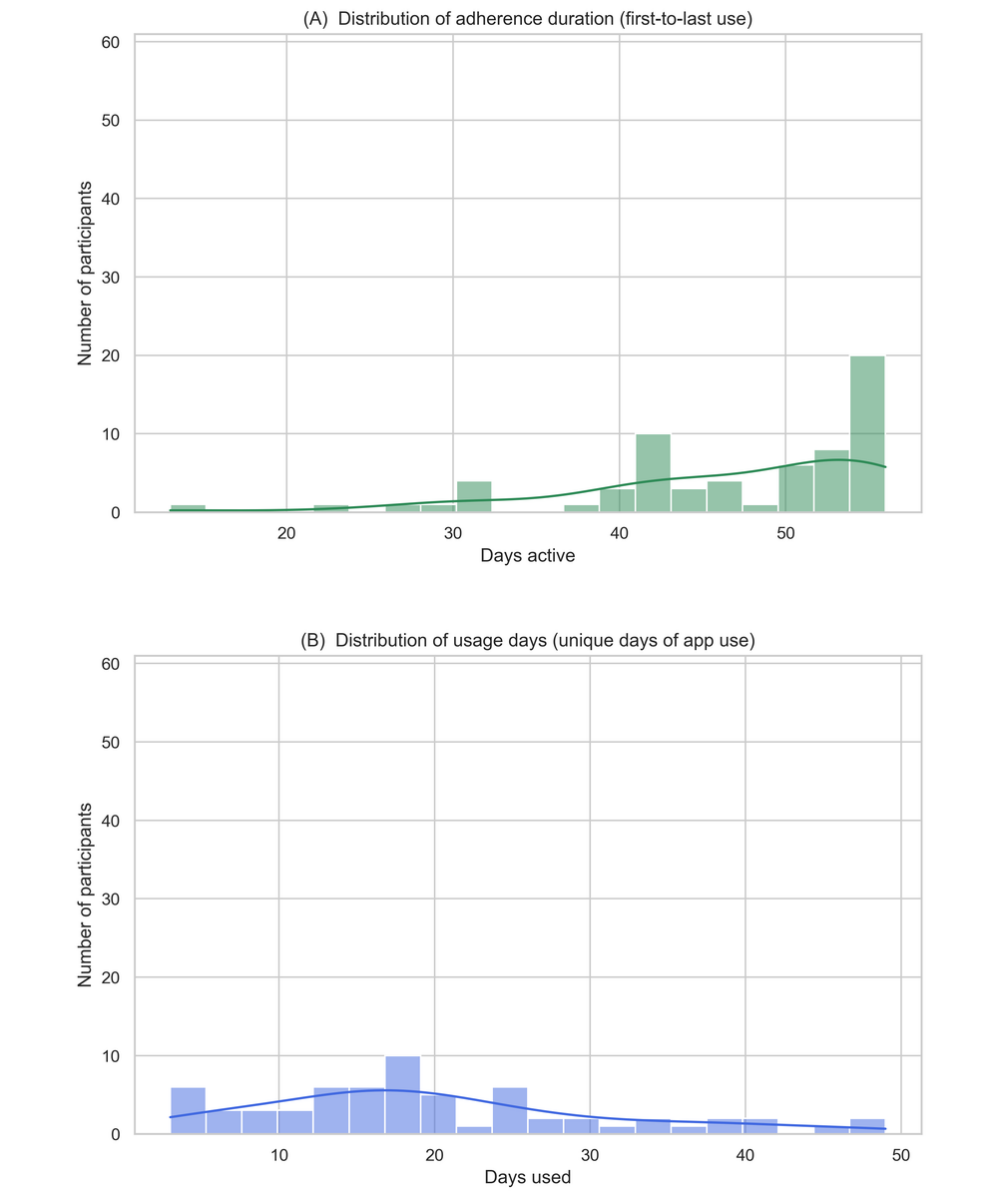
Platform use. (A) Adherence and (B) usage.

Adherence to platform use was defined as the proportion of days between the first and last app use relative to the individual study period, whereas app usage referred to the total number of distinct days on which the app was used (Figure 3).

**Table 5:** Users’ sociodemographic characteristics.

Sociodemographic characteristics		Older adults (n=61)	Caregivers (n=17)
Age, mean (SD)		73.9 (6)	47.5 (9.8)
**Gender, n (%)**			
	Male	24 (39)	10 (59)
	Female	37 (61)	7 (41)
**Marital status, n (%)**			
	Married	36 (59)	5 (29)
	Full-time relationship	2 (3)	4 (24)
	Separated	3 (5)	N/A^a^
	Divorced	4 (7)	N/A
	Single	2 (3)	8 (47)
	Widowed	14 (23)	N/A
**Education, n (%)**			
	No education	1 (2)	N/A
	Primary	5 (8)	2 (12)
	Secondary	27 (44)	6 (35)
	Tertiary	28 (46)	9 (53)
**Residence, n (%)**			
	Urban area	54 (89)	16 (94)
	Rural area	7 (11)	1 (6)
**App use, median (IQR)**			
	Adherence (%)	85.6 (74.1-96.8)	N/A
	Usage (days)	18.0 (13.0-24.2)	N/A

^a^N/A: not applicable.

### Primary Outcomes

#### Usability

The average SUS score among all older end users was 68, corresponding to the benchmark 50th percentile. More specifically, the mean SUS score of the primary users was 67.62. According to adjective ratings typically associated with SUS scores, this result would be classified as “OK,” suggesting that although the system was generally usable, there remains room for improvement. The lowest SUS scores were observed in Austria (50.83 at T1 and 45.94 at T2), whereas the highest scores were observed in Romania (74.46 at T1 and 75.54 at T2). Among secondary users (caregivers), the average score was 66.3, which was also close to the “OK” range but still indicated room for improvement. Further details are provided in Table 6.

**Figure 4:**
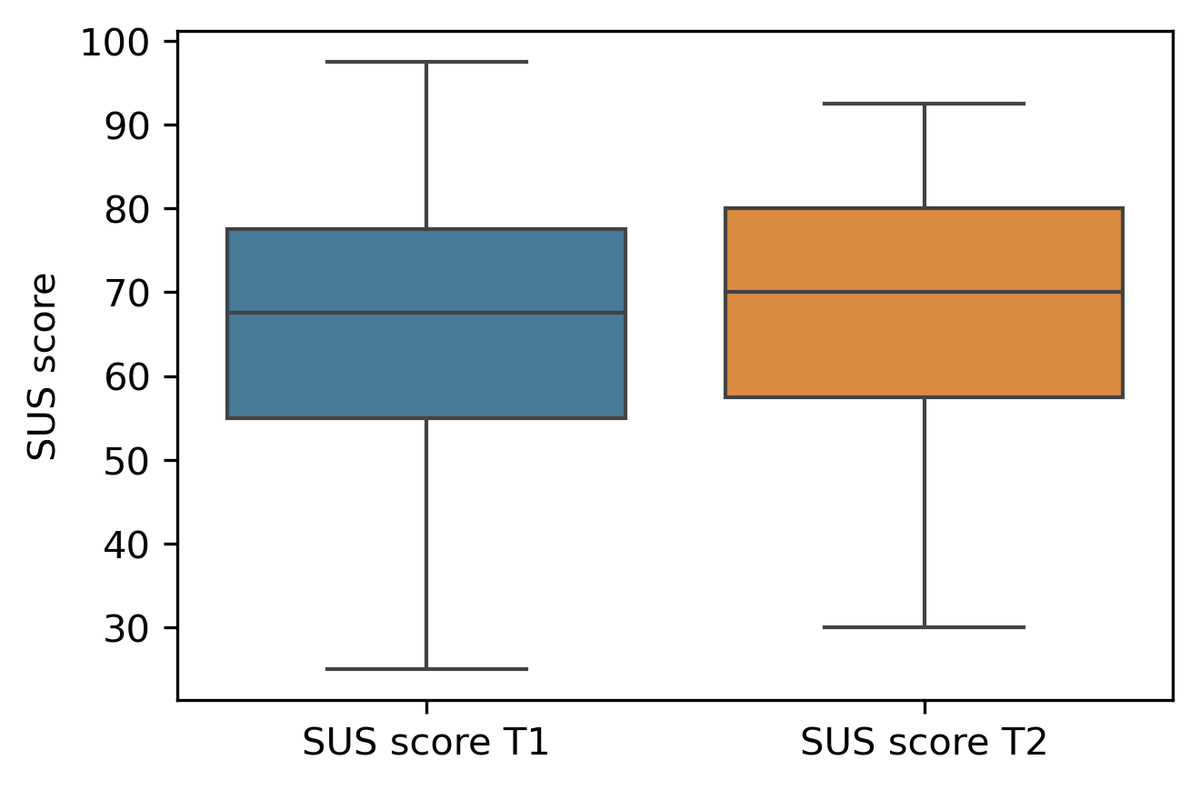
Boxplot of System Usability Scale (SUS) score at T1 and T2.

As shown in Figure 4, the SUS scores had median values of 67.50 (IQR 22.50) at T1 and 70.00 (IQR 22.50) at T2. The Wilcoxon signed rank test indicated no significant difference between the 2 time points (*P*=.42).

**Table 6:** System Usability Scale and User Experience Questionnaire short version scores among older adults and caregivers.

Scales and their scores				Older adults (n=61), mean (SD)		Caregivers (n=17), mean (SD)
**System Usability Scale** **score**				67.62 (15.09)		66.2 (21.25)
	**Nation**					
		Austria (n=8)	45.94 (9.99)		N/A^a^	
		Italy (n=30)	67.33 (13.06)		N/A	
		Romania (n=23)	75.54 (11.28)		N/A	
	**Gender**					
		Female	70.34 (14.51)		N/A	
		Male	63.44 (15.30)		N/A	
	**Education level**					
		Primary or no education	57.08 (14.53)		N/A	
		Secondary education	66.96 (15.65)		N/A	
		Tertiary education	70.65 (13.95)		N/A	
**User Experience Questionnaire short version** **score**						
	Hedonic Quality		1.66 (0.90)		1.22 (1.39)	
	Pragmatic Quality		1.23 (1.02)		0.76 (1.31)	
	Overall		1.45 (0.83)		0.99 (1.27)	

^a^N/A: not applicable.

#### User Experience

As reported in Table 6, the overall UEQ score among primary users was 1.45, suggesting a “good” overall user experience in which both functional and emotional needs were reasonably well met.

A high Hedonic Quality score of 1.66 indicated that the product was perceived as enjoyable, stimulating, and innovative. This emotional appeal enhanced user satisfaction and could promote greater engagement with the product.

A Pragmatic Quality score of 1.23 reflected above-average usability, indicating that the product was generally effective and supportive for task completion, although there was still room for improvement in areas such as clarity and efficiency.

As reported in Table 6, the overall UEQ score among secondary users was 0.99, suggesting a generally positive experience. A Hedonic Quality score of 1.22 indicated that users found the product somewhat engaging and emotionally appealing, although there was room for improvement to make it more exciting or innovative. Moreover, a Pragmatic Quality score of 0.76 suggested below-average usability, indicating that users experienced challenges related to efficiency, clarity, or task support.

**Figure 5:**
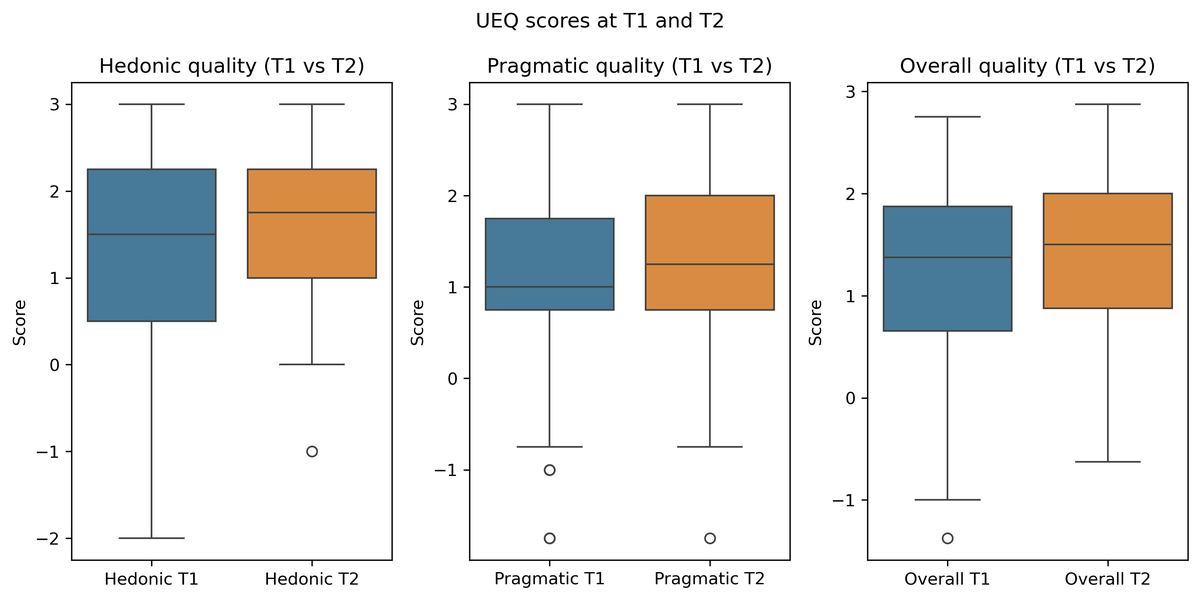
Boxplots comparing the distribution of User Experience Questionnaire (UEQ) scores at 2 time points (T1 and T2). The subplots present hedonic quality, pragmatic quality, and overall experience scores, showing a general increase from T1 to T2. This trend is consistent with statistically significant improvements across all dimensions (hedonic quality: P=.003; pragmatic quality: P=.04; overall experience: P=.001).

As shown in Figure 5, the UEQ scores demonstrated statistically significant improvements from T1 to T2 across all evaluated dimensions. Hedonic Quality increased, with median values rising from 1.50 (IQR 1.75) at T1 to 1.75 (IQR 1.25) at T2 (*P*=.003). Pragmatic Quality improved from a median of 1.00 (IQR 1.00) to 1.25 (IQR 1.25) (*P*=.04). Overall experience also improved significantly, with median values increasing from 1.38 (IQR 1.25) to 1.50 (IQR 1.12; *P*=.001). These distributions are visually summarized by boxplots in Figure 5, illustrating both the positive trend and the distribution of scores at each time point.

#### Acceptance

Primary users reported a generally positive perception of the CAREUP platform, describing it as interesting, useful, and efficient (n=49). Frequently appreciated features included ease of use and innovative technology (n=25), health monitoring (n=20), games (n=5), the smartwatch for promoting physical activity (n=4), and the mattress sleep-tracking function (n=3).

Key issues raised included the excessive number of devices (n=7), unmet expectations over time (n=5), time-consuming technical and connectivity problems (n=20), game-related issues (n=5), repetitive questionnaires (n=5), overload caused by additional devices (n=3), and concerns regarding the reliability of the results (n=1).

Regarding recommendations, 48 participants stated that they would endorse the platform to a friend, 6 would recommend it only to individuals with strong technical skills, 3 would recommend it if improvements were made, 2 were undecided, and 2 would not recommend it. Regarding willingness to continue using CAREUP, 39 participants agreed, 8 stated they would use it occasionally, 2 would continue if fewer devices were required, 1 was undecided, and 11 declined.

Regarding the system’s impact on daily routines, CAREUP improved daily routine management for 34 users, whereas 26 disagreed and 1 was undecided. CAREUP did not interfere with daily routines for 38 users; however, 21 reported interference, and 2 reported occasional interference.

Suggestions for new users included frequent use to become familiar with the system (n=17), active participation because the platform was perceived as simple and useful (n=16), maintaining confidence and patience (n=10), becoming familiar with the technology and reading the manual beforehand (n=7), seeking help when needed (n=4), and following the provided instructions (n=3). Additional comments included requests for game enhancements (n=6), technical improvements (n=4), fewer devices (n=3), additional training or manuals to address system complexity (n=3), more exercises (n=3), clearer instructions or instructional videos (n=2), and dietary advice (n=1).

Secondary users identified the following as the best aspects of the platform: continuous health monitoring (n=8), data storage (n=10), games (n=4), integration of apps and devices (n=4), the smartwatch for promoting physical activity (n=2), and the sense of providing care and support (n=1).

Key criticisms included system complexity (n=5); difficulties in managing health results (n=2); unclear graphs (n=2); detection errors, technical issues, or a confusing interface (n=9); monitoring being perceived as a form of control (n=1); the short trial duration (n=2); and the need for prior familiarity with technology (n=1).

Regarding recommendations to other caregivers, 8 participants stated that they would endorse CAREUP, 1 would recommend it partially, 3 were undecided, and 4 would not recommend it.

Regarding willingness to continue using CAREUP, 6 participants agreed, 6 were undecided, and 5 declined.

Regarding interference with the care recipients’ daily routines, CAREUP was reported not to interfere by 10 participants, to interfere by 3 participants, whereas 4 participants were undecided.

Suggestions for new users included being patient while learning to use the system (n=8), assessing older adults’ interest in the platform (n=3), and maintaining real interpersonal relationships alongside technology use (n=1).

Experience-based comments highlighted the need to simplify the system and address technical issues (n=3).

All opinions regarding acceptance are summarized in Multimedia Appendix 3.

### Secondary Outcomes

#### Data Overview

All data related to the secondary outcomes are reported in Table 7.

**Table 7:** Secondary outcomes.

Test					Respondents, n			Physical component summary at T0, mean (SD)			Physical component summary at T2, mean (SD)			*P* value
**Physical component summary**					61			46.35 (7.63)			45.88 (7.97)			.75
	**Nation**													
		Austria	8			48.67 (5.29)			49.02 (5.95)			.95		
		Italy	30			49.47 (6.29)			48.50 (7.46)			.76		
		Romania	23			41.28 (7.62)			41.11 (7.32)			.71		
		Gender												
		Female	37			45.54 (8.01)			44.89 (8.77)			.71		
		Male	24			47.59 (7.00)			47.39 (6.45)			.96		
**Mental component summary**					61			49.74 (8.56)			51.37 (9.02)			.15
	**Nation**													
		Austria	8			49.58 (8.08)			52.19 (8.10)			.57		
		Italy	30			48.30 (8.84)			48.30 (10.08)			.85		
		Romania	23			51.70 (8.37)			55.00 (6.43)			.03		
	**Gender**													
		Female	37			48.76 (9.53)			50.97 (10.51)			.20		
		Male	24			51.24 (6.75)			51.96 (6.25)			.76		
**Instrumental Activities of Daily Living**					61			7.75 (0.98)			7.84 (0.64)			.64
	**Nation**													
		Austria	8			8.00 (0.00)			8.00 (0.00)			>.99		
		Italy	30			7.93 (0.37)			7.93 (0.37)			.37		
		Romania	23			7.43 (1.50)			7.65 (0.93)			.23		
	**Gender**													
		Female	37			7.81 (1.00)			7.92 (0.36)			.37		
		Male	24			7.67 (0.96)			7.71 (0.91)			.72		
**UCLA version 3**					61			53.51 (4.88)			51.60 (10.36)			.17
	**Nation**													
		Austria	8			54.80 (3.26)			43.90 (23.28)			.21		
		Italy	30			53.53 (4.58)			52.93 (4.14)			.23		
		Romania	23			52.91 (5.82)			53.22 (5.07)			.81		
	**Gender**													
		Female	37			53.74 (5.47)			51.89 (9.87)			.19		
		Male	24			53.16 (3.89)			51.16 (11.25)			.52		
**Lubben Social Network Scale—Revised**					61			34.14 (7.87)			33.62 (10.29)			.50
	**Nation**													
		Austria	8			34.10 (7.89)			26.10 (15.18)			.23		
		Italy	30			32.77 (7.70)			33.60 (9.69)			.32		
		Romania	23			35.96 (8.07)			36.91 (6.58)			.09		
	**Gender**													
		Female	37			34.21 (7.81)			34.26 (9.75)			.95		
		Male	24			34.04 (8.14)			32.64 (11.19)			.36		
**15-item Geriatric Depression Scale**					61			2.51 (2.14)			2.26 (1.89)			.17
	**Nation**													
		Austria	8			2.25 (1.75)			1.88 (1.73)			.17		
		Italy	30			2.50 (2.00)			2.67 (2.14)			.76		
		Romania	23			2.61 (2.50)			1.87 (1.52)			.002		
	**Gender**													
		Female	37			2.65 (2.29)			2.35 (1.89)			.34		
		Male	24			2.29 (1.92)			2.12 (1.92)			.18		
Visual Analogue Scale					61			77.67 (12.55)			79.30 (9.75)			.29
Montreal Cognitive Assessment					61			27.68 (2.29)			27.75 (2.29)			.16

#### SF-12 v2

The results obtained from the primary users showed that the mean physical component summary scores remained overall stable over time, with values slightly below the standardized population mean of 50. The slight decrease observed at T2 did not appear to be clinically significant. In addition, women showed lower mean scores than men at all time points, indicating poorer physical health status. Men maintained stable scores over time, with values close to the standardized mean of 50, whereas women showed a slight decline at T2. Regarding the mental component summary score, the mean value improved slightly over time, from 49.74 at T0 to 51.37 at T2. The values remained close to the standardized population average of 50, indicating a mental health status consistent with the population average. Whereas the scores for men remained stable across the 3 assessments, women showed a slight improvement in mental well-being from T0 to T2.

#### IADL

The IADL (Instrumental Activities of Daily Living) scores of the primary users were very close to the maximum value of 8, indicating that participants were largely independent in their daily activities. Although both women and men demonstrated a high level of independence, women showed higher scores across all 3 assessments.

#### UCLA v3

Scores on the loneliness scale (UCLA Loneliness Scale) showed a slight decrease over time. Overall, the scores remained within the moderate-to-high loneliness range, with a trend toward slight improvement in the social and emotional well-being of the participants (primary users). However, the results showed no significant differences according to gender (*P*=.63) or country (Austria, *P*=.21; Italy, *P*=.23; and Romania, *P*=.81).

#### LSNS-R

Data analysis showed that the LSNS-R scores of the primary users fell within the 31-44 range on the LSNS scale, indicating moderate social support for the group as a whole. This finding suggested that most participants had a satisfactory social support network, although a slight decline was observed over time. No significant differences were observed between women and men (Austria, *P*=.23; Italy, *P*=.32; and Romania, *P*=.09).

#### GDS-15

All mean GDS-15 scores (T0 and T2) were below 5, indicating that most participants did not have significant depressive symptoms. No significant differences were observed between women and men (*P*=.79).

#### EQ-5D-5L

Analysis of the EQ-5D-5L results showed that more than half of the sample reported no problems with mobility, self-care, or performing usual activities. Approximately half of the sample reported slight physical pain, and nearly half reported slight anxiety. The Visual Analogue Scale component of the assessment, which evaluated perceived health status, indicated a generally positive perception of health.

#### MOCA

The mean MOCA scores of the primary users were 27.68 at T0 and 27.75 at T2, indicating good cognitive function among the participants.

#### Game Experience Questionnaire, Part 3 Postgame Module

The postgame module’s positive experience score of 1.55 suggested that participants were able to play the games without difficulty. No negative experiences, tiredness, or issues related to returning to reality were reported. Further details are presented in Table 8.

**Table 8:** Postgame module scores.

Game Experience Questionnaire score	T1, mean (SD)	T2, mean (SD)	*P* value
Positive experience	1.55 (1.11)	1.56 (1.08)	.65
Negative experience	0.39 (0.74)	0.35 (0.67)	.55
Tiredness	0.18 (0.41)	0.15 (0.36)	.56
Returning to reality	0.44 (0.54)	0.40 (0.52)	.59

#### Cares Workload

##### Overview

The secondary users (caregivers) did not report any notable changes in caregiving workload.

##### Devices Used During the Pilot

Users utilized the system devices at varying frequencies across different device types and pilot countries. The least-used component was the PH app, which was accessed less than once per week in both Italy and Romania. This finding highlights the importance of tailoring the positive health questionnaires to better address key aspects of older adults’ social and psychological well-being. On average, the devices were used 2 times per week. The most frequently used devices were those commonly adopted by older adults, such as the oximeter and blood pressure monitor, followed by the bathroom scale. Several users engaged with these devices very actively, with 1 participant from Romania recording more than 200 weight measurements during the course of the pilot. The glucose meter and hand-grip strength meter were used less frequently. Mattress sleep sensors were used only in Italy. Their average use was approximately 3 times per week, which may have reflected periods during which the devices were unplugged or used for only part of the pilot duration.

Component usage varied across countries. The PH app was used more frequently in Austria, whose population is generally more digitally literate than those of Italy and Romania. Device use was generally similar across countries; however, users in Austria used the devices less actively than users in Italy and Romania. The greatest difference in usage frequency was observed for the hand-grip dynamometer, which was rarely used in Romania. These differences indicate the importance of considering users’ digital literacy and preferences when introducing novel technological solutions. Table 9 provides detailed information on weekly system device use across device types and pilot countries.

**Table 9:** System devices use intensity in times per week.

Device and nation			Mean (SD)		Median		Range	
**Positive Health app**								
	Austria	1.47		1.50 (0.90)		0.28-2.92		
	Italy	0.45		0.66 (0.77)		0.00-3.89		
	Romania	0.51		0.65 (0.50)		0.08-2.14		
**Glucose meter**								
	Austria	N/A^a^		N/A		N/A		
	Italy	0.80		0.87 (0.49)		0.15-1.69		
	Romania	0.96		1.29 (1.39)		0.10-4.98		
**Oximeter**								
	Austria	1.56		1.82 (0.95)		0.49-3.15		
	Italy	2.75		2.83 (1.47)		0.60-6.70		
	Romania	1.69		2.80 (2.66)		0.16-9.48		
**Bathroom scale**								
	Austria	1.47		1.73 (1.16)		0.37-3.68		
	Italy	1.72		2.10 (1.53)		0.22-6.50		
	Romania	1.37		2.64 (4.03)		0.10-19.49		
**Hand-grip strength meter**								
	Austria	2.03		1.77 (0.96)		0.14-2.93		
	Italy	2.07		2.46 (1.47)		0.74-6.40		
	Romania	0.16		0.24 (0.21)		0.10-0.60		
**Blood pressure meter**								
	Austria	1.30		1.69 (1.38)		0.00-3.41		
	Italy	2.45		2.50 (1.32)		0.22-5.91		
	Romania	1.93		2.77 (2.64)		0.10-8.42		

^a^N/A: not applicable.

## Discussion

### Principal Findings

The CAREUP pilot feasibility study evaluated the usability, user experience, and acceptance of an integrated care platform for tracking the IC of older individuals using various monitoring devices. Secondary outcomes included assessments of physical and mental health, quality of life, social interaction, and caregiver workload.

The CAREUP platform demonstrated both successes and challenges during the study. Although participant numbers varied across countries, no direct correlation was found between country and SUS scores. Differences in SUS ratings appeared to be more closely related to factors such as device connectivity, interface clarity, and users’ digital literacy levels than to cohort size [60]. These findings suggest that local contextual and technical conditions have a stronger impact on usability perceptions than the number of participants at each site. Digital skills vary among individuals according to their level of autonomy in using technology and are influenced by socioeconomic status [61]. Specifically, Austria’s lower scores highlighted issues with device connectivity and interface clarity that remained largely unresolved by the end of the trial. By contrast, Romania’s favorable scores suggested that the platform could be effective and well-received when it functions smoothly.

Despite these usability challenges, many older adults (the primary users) viewed CAREUP as engaging, innovative, and empowering. Adopting a technological health solution can initially pose varying degrees of difficulty, depending on an individual’s prior experience and available resources [62]. Nonetheless, most people are willing to learn and adopt new digital tools if they perceive tangible benefits for their health and well-being [63]. User experience assessments reflected a predominantly positive outlook, especially regarding the platform’s “hedonic qualities”—its emotional appeal. Participants valued access to cognitive games and health-monitoring features, which introduced stimulation and novelty into their daily routines. Nevertheless, some critical feedback was noted.

Acceptance among older adults and caregivers revealed a nuanced picture. Many users expressed enthusiasm for CAREUP, appreciating its capacity to track health parameters and support proactive health management. However, persistent technical obstacles—such as connectivity issues between devices and software—tested participants’ patience. Both groups also found the number of required devices cumbersome. Although health technologies are becoming increasingly accepted in older adults’ lives, partly accelerated by the COVID-19 pandemic [64], a digital divide remains, contributing to divergent views on acceptance. Although older adults typically acknowledge the benefits of technology for improving care efficiency and quality, they often struggle with adaptation. Therefore, promoting digital literacy is crucial for fostering their engagement and inclusion in the digital health care landscape [65].

As secondary users, caregivers brought a nuanced perspective to CAREUP. They valued its ability to reduce worry through continuous health monitoring and to provide structured insights into their loved ones’ health. However, many also found the system unnecessarily complex. This tension between appreciation and frustration highlighted the need for targeted refinements, particularly simplifying the platform and improving reliability. For digital solutions to succeed, it is vital to involve all key end users—patients, caregivers, and health care professionals—early in development [66]. Caregivers, in particular, play a pivotal role; thus, it is essential not only to engage them but also to provide sufficient digital training. Empowering caregivers in this way enables them to support older users more effectively and substantially increases the accessibility and success of digital solutions [67]. More broadly, the CAREUP pilot exemplified both the challenges and opportunities associated with introducing technology into complex, human-centered settings. Technical reliability emerged as a major challenge, repeatedly undermining the user experience. Frequent Bluetooth issues and software glitches frustrated users and, at times, alienated them from the platform. This underscores the cost of technological shortcomings: regardless of innovation, systems must function seamlessly to earn users’ trust and loyalty. Poor reliability can foster frustration, leading to abandonment and an increased risk of digital exclusion [68]. Multiple factors influence the use of digital technology, and disengagement may stem from psychological reasons (such as low motivation, lack of confidence, or perceived lack of usefulness), physical barriers (such as limited access), or practical challenges (including insufficient digital skills) [69].

The issues described are also common in other platforms evaluated in usability studies reported in the literature. Issues such as connectivity problems leading to delayed data availability [22] and difficulties understanding how to use specific components [23,27,33] are particularly prevalent in novel solutions. Interface aesthetics are also a commonly raised concern, with users often requesting personalization options to better tailor the solution to their preferences [70].

Another key takeaway from the pilot was the importance of user education and support. Although digital technologies are a valuable resource in health care, older adults still lag significantly behind younger individuals in their use of these technologies [71]. Their limited digital health literacy is influenced by both subjective factors—such as anxiety, stress related to technology use, and lack of confidence—and objective factors [72]. These include sociodemographic factors (eg, age, education level, and economic status), digital device-related factors (eg, internet access and usability challenges), and social support factors (eg, support provided by family members) [73].

Even though each pilot site organized an instructional phase at the beginning of the pilot and provided a user manual, some participants, especially those less familiar with technology, struggled to fully understand the platform’s capabilities. This was particularly evident during the early phase of the trial, when users were still acclimating to the system. User comments also highlighted the potential value of video tutorials or in-person training for enhancing familiarity and confidence with the system. This may serve as a recommendation for future implementation. Given the persistent gap between the availability of digital health solutions and older adults’ ability to use them effectively, ensuring adequate digital health literacy is essential [74]. To provide tailored support, it is important to first assess users’ level of digital literacy, specifically their ability to search for, navigate, understand, and evaluate digital health information and tools [75].

### Limitations, Implications, and Future Directions

#### Pilot and Implementation Considerations

This study has several limitations inherent to pilot research and the implementation of digital health interventions:

#### Sample Size

The relatively small sample size (n=66 enrolled; n=61 completed) limits statistical power and the generalizability of the findings. As this was an exploratory pilot study, no formal sample size calculation was performed; therefore, the results should be interpreted as formative and hypothesis-generating rather than confirmatory. The small sample size, coupled with gender imbalance and the specific national contexts of Italy, Romania, and Austria, introduces potential bias and further constrains the generalizability of the findings. These demographic and cultural differences should be considered when interpreting the results.

#### Selection Bias

Recruitment was conducted at 3 sites using nonrandom convenience sampling. This may have resulted in a study population that was more motivated or technologically skilled than the general older adult population, thereby introducing potential bias.

#### Missingness

Participant dropout and incomplete app or device use contributed to missing data, particularly for secondary outcomes that relied on self-report and device connectivity.

#### Device and Data Constraints

Usability challenges, technical difficulties related to device connectivity, and variability in digital literacy affected the consistency and completeness of data collection. The short intervention duration and reliance on self-reported or device-captured metrics further limit the robustness of the clinical inferences.

#### Technology Readiness Level

Technology Readiness Level (TRL) may have influenced the results. Before the start of the pilots, the platform components (apart from the off-the-shelf sensors) had been tested only in a laboratory or simulated operational environment (TRL 5 and TRL 6). Despite the relatively low TRL, our approach is consistent with current research standards in the field [76], which identified numerous studies in this area that remain in the development and testing phase (TRL 5) or in the demonstration, pilot, and prototypical system validation phase (TRL 6).

#### Mitigation and Future Directions

To address these limitations, subsequent phases of the CAREUP project will target a larger and more diverse multicenter sample and implement randomized recruitment methods where feasible. Platform development will include expanded modularity and adaptive features to support broader usability and reduce technological barriers. Clinical end points (eg, hospitalization and independent living status) and longer follow-up periods will be incorporated to capture outcomes with greater relevance and reliability. Technical upgrades, enhanced user support, and expanded device interoperability are also planned.

A comprehensive impact evaluation is scheduled within the next 18-24 months, with interim analyses and publication of progress updates at 12 months to guide iterative refinement and reporting.

Sharing both the strengths and limitations of research is fundamental to enabling more effective advancement in the field. Although new technologies increasingly demonstrate substantial utility in supporting health maintenance, self-care, independence, and autonomy—all of which contribute to improvements in quality of life—current approaches remain limited in scope.

Looking ahead, the CAREUP project revealed several valuable lessons for future development. First, robust testing and debugging should be prioritized to ensure the reliability of devices and software. Second, the platform’s interface should be simplified, with clearer pathways and visual guidance to make navigation intuitive for a broad demographic. Third, reducing the number of devices or integrating functionalities could address complaints about device overload and improve user-friendliness. Finally, cultural and contextual differences, as reflected in the varied reception across countries, should inform future adaptations to ensure that the platform resonates with diverse user groups. Relevant information was continuously shared with the technical partners to support integration and development of the advanced prototype and final product.

### Conclusions

The CAREUP pilot study demonstrates the potential of integrated digital health platforms to support healthy aging through IC monitoring. Despite the challenges associated with introducing technology into human-centered health care, the platform’s positive outcomes highlight the promise of digital health solutions for the future. The study also underscores the importance of cultural adaptation in digital health interventions, demonstrating that optimal adoption of multidimensional health-monitoring technologies among older adults requires attention to technical reliability, user interface design, and cultural context. Variations in usability scores across countries further emphasize this need.

Technical reliability is essential for user acceptance, as connectivity issues and software glitches can undermine the user experience and lead to platform abandonment.

However, system complexity and the need for digital literacy support underscore the importance of engaging all stakeholders—patients, caregivers, and health care professionals—early in the development process.

Future implementations of digital health platforms for aging populations must address technical infrastructure, user-friendly interfaces, comprehensive education, cultural adaptation, and multidimensional health monitoring. By meeting these requirements, platforms such as CAREUP can more effectively support healthy aging and enhance the quality of life of older adults and their caregivers.

The pilot also highlighted that technology-based interventions often focus on single domains (physical, cognitive, mental, emotional, etc) rather than adopting a multidimensional approach to health promotion. Expanding research in this direction could substantially strengthen the evidence base and further advance the field.

## Appendix

Multimedia Appendix 1 Checklist of iCHECK-DH (Guidelines and Checklist for the Reporting on Digital Health Implementations) guidelines.

Multimedia Appendix 2 Careup Positive Health app question selection cycle. Questions selected for each 4-week cycle day are marked with an “x.” Question identifiers correspond to the labels used in the CAREUP pilot dataset.

Multimedia Appendix 3 Acceptance rating among primary users.

## Abbreviations

GDS-15: 15-item Geriatric Depression Scale
IADL: Instrumental Activities of Daily Living scale
IC: intrinsic capacity
iCHECK‑DH: Guidelines and Checklist for the Reporting on Digital Health Implementations
INRCA: Istituto Nazionale di Ricovero e Cura per Anziani
LSNS-R: Lubben Social Network Scale—Revised
MOCA: Montreal Cognitive Assessment
PH app: Positive Health app
REST: Representational State Transfer
SF-12: 12-Item Short Form
SUS: System Usability Scale
TRL: Technology Readiness Level
UEQ-S: User Experience Questionnaire short version
