# Supplementary material for: A Multidimensional Digital Health Platform to Support Intrinsic Capacity and Healthy Aging: Pilot Feasibility Study
Source: JMIR Aging. 2026 Jul 28;9:e79862. doi: 10.2196/79862 (PMC13462729; doi:10.2196/79862)
Supplement: Supplementary file 1 [file aging_v9i1e79862_app1.docx]

**Multimedia Appendix 2.** Checklist of iCHECK-DH guidelines.

| Section |  | Item | Description |
| --- | --- | --- | --- |
| Title | 1 | Title  (M^[1]^) | A Multidimensional Digital Health Platform to Support Intrinsic Capacity and Healthy Aging: Usability, Acceptance, and Impact in a Multicenter European Pilot Study |
| Abstract | 2 | Abstract  (M) | The CAREUP pilot evaluated a digital health platform for monitoring and supporting Intrinsic Capacity (IC) in older adults across Italy, Romania, and Austria. The implementation used a multicenter strategy, enrolling adults aged 65+ and caregivers, and integrated wearable sensors and mobile apps for real-time tracking of physiological, cognitive, and behavioral health. Feasibility, usability, and acceptance were measured using the System Usability Scale and User Experience Questionnaire, with support provided through training and helpdesk services.  Key implementation elements included personalized dashboards, modular architecture, data privacy protections, and local onboarding. Primary outcomes tracked were user experience, adherence, and data completeness; secondary health outcomes included trends in physical, mental, and social well-being. KPIs/outputs comprised completion rates, usability scores, health status changes, and qualitative feedback on barriers. The study established foundational insights for future scale-up and is registered on ClinicalTrials.gov (NCT05963399) |
| Introduction | 3 | Context  (M) | The CAREUP pilot was conducted in Italy, Romania, and Austria, engaging urban and rural health centers, research institutes, and community partners focused on older adults and their caregivers. Implementation faced challenges such as variable digital literacy, resource disparities, and healthcare fragmentation—especially in Italy and Romania—while Austria benefited from stronger eHealth infrastructure and policy support. The study targets independent older adults (≥65) and leverages multidisciplinary teamwork for training and support. Barriers included digital skills gaps and fragmented policy environments, while facilitators were strong local partnerships and alignment with Austria’s advanced national digital health strategy; alignment is partial in Italy and Romania, which are still developing robust digital health frameworks. Currently, CAREUP is at the “Piloting and Evidence Generation” stage, with plans for scale-up after demonstrating feasibility and formative impact..^[2]^ |
|  | 4 | Problem statement  (M) | The CAREUP pilot is a multicenter digital health implementation focused on supporting older adults’ healthy aging through self-monitoring of Intrinsic Capacity using wearable sensors and mobile applications. The platform integrates physiological, cognitive, and behavioral data streams delivered via a secure cloud infrastructure. The study engaged community-dwelling adults aged 65+ and their caregivers across Italy, Romania, and Austria. It evaluates feasibility, usability, and preliminary health outcomes, emphasizing user empowerment and tailored support. The implementation addresses fragmented, episodic traditional care by promoting proactive, integrated, and personalized health management to improve quality of life among seniors. This foundational work informs larger scale deployment and aligns with evolving European digital health strategies |
|  | 5 | Similar Interventions  (M) | The CAREUP platform was inspired by previous digital health monitoring solutions for older adults and similar platforms that combine wearable sensors, health questionnaires, and personalized dashboards for self-monitoring. CAREUP’s added value lies in its multidimensional approach—explicitly targeting Intrinsic Capacity (IC) across multiple domains (physical, cognitive, psychological, social)—and providing predictive, adaptive self-care recommendations. Unlike many predecessors, CAREUP integrates commercial and custom devices, cognitive games, and tailored care plans using a user-centered, co-design process involving seniors, caregivers, and multidisciplinary partners from inception.  Key differences include:   - Stronger focus on IC as the organizing framework, beyond just vital signs or activity. - Gamification and regular feedback to drive adherence and engagement. - Extensive cross-national adaptation, testing in three European contexts, and iterative local support for technology onboarding. - Prioritization of both the end-user (older adult) and the secondary user (caregiver) experience, with formal assessment of usability and acceptance. - Technical architecture designed for modular expansion, data privacy, and compatibility with future health record integration.   Compared with existing models, CAREUP offers a more comprehensive, adaptive, and inclusive solution for older adults’ self-management and sustainable aging |
| Methods | 6 | Aims and Objectives  (M) | The CAREUP pilot’s main objective was to test the feasibility, usability, and preliminary health impact of a digital health platform designed for self-monitoring and personalized support of older adults’ physical, cognitive, and behavioral health. Impact was measured primarily by user experience and retention (using SUS and UEQ-S scores and completion rates), and secondarily by changes in independence, cognitive function, mental health, and quality of life (using validated scales like SF-12, IADL, MOCA, and EQ-5D-5L). Key performance indicators included usability and satisfaction ratings, participant retention, system usage, and stability or improvement in health status over the study period. |
|  | 7 | Blueprint summary  (M) | The implementation strategy centered on co-design with end users, local adaptation, and robust onboarding:   - Participants received in-person training, user manuals, and ongoing tech support. - Device usage was tailored to individual capabilities and preferences, reducing complexity and maximizing adherence. - Data collection occurred at three time points during two months (baseline, midterm, final), with quantitative and qualitative assessments for system usability, health outcomes, and user experience. - Technical and organizational barriers were addressed with real-time assistance and iterative feedback to developers.   The roadmap prioritizes iterative improvement, including interface simplification, expanded device interoperability, enhanced training materials, and planning for scale-up and integration with national health data systems. Early stakeholder engagement, continuous adaptation, and future technical upgrades are central to sustaining impact and broader uptake |
|  | 8 | Technical Design  (M) | The platform integrates Android apps (for care plans and health assessment), commercial and custom wearable devices (blood pressure, weight, oximetry, step counts), and cognitive games. These connect to a cloud-based dashboard for visualization and feedback, with data uploaded securely via REST APIs. CAREUP is modular, supporting future expansion and planned FHIR/HL7 interoperability for health system integration and research.  Technology features include AI-driven real-time health monitoring, personalized predictive feedback, and user empowerment through visualization and gamification. Licensing is mixed: commercial device hardware/software, custom-developed open-source code and proprietary modules (with consortium IP ownership), and research-use licensing for study applications. The platform is not fully open source but uses open standards for interoperability; code documentation and the application are available via the consortium’s repository with public access for research (e.g., Zenodo dataset) and more info on the project website. |
|  | 9 | Target  (M) | The target of the CAREUP intervention was community-dwelling older adults (≥65 years) in Italy, Romania, and Austria, who lived independently, had cognitive capacity, and were willing to provide informed consent. Caregivers (mainly informal) participated as secondary users. Site selection required centers able to support digital intervention deployment, with trained staff, technical resources, and willingness to adapt procedures to local contexts. Sites included both urban and rural facilities, ensuring exposure to diverse digital literacy levels and social contexts.  Eligibility criteria for participants included age ≥65, independent living, ability and willingness to consent, no acute or severe health contraindications, and absence of incompatible medical devices. Exclusion criteria encompassed complex comorbidities, severe sensory or cognitive impairment, or recent acute events. Caregivers did not have specific inclusion/exclusion criteria. Implementing sites had to provide on-site training, technical set-up for devices/apps, and ongoing support, while fostering a user-centered and adaptive environment for both older adults and care teams |
|  | 10 | Data  (M) | CAREUP followed robust data governance based on GDPR-compliance and “security by design” principles. Data collection involved secure acquisition of physiological, behavioral, and cognitive info via sensors, apps, and self-reports. Data were pseudonymized, encrypted, transmitted via secure REST APIs, and hosted on a cloud-based platform located within the EU, meeting in-country/European requirements. Only authorized researchers and clinical partners accessed data using role-based credentials; patients and caregivers had user-specific dashboard access to their health results and activity logs. All research participants gave written, informed consent at enrollment—this covered data use, confidentiality, and publication of anonymized results.  Routine data are kept confidential and not linked to personal identifiers. Aggregated de-identified data may be shared for research or cross-national analysis per project protocols and participant consent. Data access and sharing policies comply with GDPR, including data minimization, right to erasure, and limitations on cross-border transfers without explicit agreement. |
|  | 11 | Interoperability  (M) | It incorporates cognitive games, validated questionnaires, and health sensors, using modular cloud-based architecture and secure REST APIs, designed for future interoperability with HL7 FHIR and semantic ontologies like ICD and SNOMED when scaling. CAREUP leverages both AI for predictive analytics and user-centered principles for usability and engagement. |
|  | 12 | Participating entities  (M) | CAREUP is a multi-country initiative aimed at developing an affordable digital health platform that supports the healthy ageing of older adults. It is led by a consortium of SMEs, research institutions, and end-user organisations, coordinated through a joint venture to ensure commercialisation and intellectual property management. The mission is to use AI and data-driven approaches for user-centred healthcare, involving both direct user organisations and government/regional health bodies, mainly for pilots and ethical approvals. The primary funding comes from the AAL Programme and business partners, with plans for continued investment after initial launch. Ownership and further development will remain with the joint venture formed by the leading business partners |
|  | 13 | Budget Planning  (M) | NA |
|  | 14 | Sustainability  (M) | NA |
| RESULTS | 15 | Coverage  (M) | European Coverage |
|  | 16 | Outcomes  (M) | CAREUP’s pilot results showed that the platform had acceptable usability (SUS 68), good user experience (UEQ-S 1.45), and high acceptance, with most older adult participants rating it helpful and easy to use. Health, independence, and cognition measures remained stable over two months, demonstrating the feasibility and acceptability of remote digital support for aging. |
|  |  | Lessons learned  (M) | Key lessons learned from CAREUP include the importance of user-centered design, robust stakeholder involvement, and adaptable training to enhance adoption and engagement. Success factors were proactive local support, involvement of caregivers, and iterative feedback loops with users and technical teams. Cultural adaptation and ongoing user education were essential, especially in countries with lower digital literacy (Italy and Romania). Implementation challenges included technical issues (device connectivity, app complexity), resistance to technology among some seniors, and variations in digital literacy.  Budget adherence was generally good. Ongoing operational costs will include licensing, cloud hosting, device replacements, user support, and interface updates.  Recommendations:   - Prioritize technical stability and simplicity for all user interfaces. - Strengthen digital skills training before and during rollout. - Engage all end-users and local staff in co-design and testing. - Plan extra resources and time for troubleshooting and end-user support. - Customize for different national contexts and readiness levels. - Adopting these strategies will improve platform uptake, satisfaction, and sustainability in diverse care settings. |
|  | 17 |  |  |
|  | 18 | Unintended consequences  (NM^[3]^) | NA |
| Discussion | 19 | Conclusion  (M) | The results highlight the value of user-centered, adaptive solutions and ongoing technical support for successful adoption.  Future implications include the need for further technical refinement, broader customization to local cultures and literacy, and deeper integration with existing health systems. CAREUP’s success supports scaling such platforms to improve self-management and preventive care among ageing populations, while also emphasising the importance of continuous evaluation, stakeholder involvement, and partnership with public health policy for long-term impact. |
| General | 20 | General  (NM) | The pilot study received full ethical approval from local and national ethics committees in thr participating countries, including governance and data privacy reviews according to GDPR and national guidelines. All participants provided written informed consent before enrollment, with protocols registered prospectively on ClinicalTrials.gov (ID: NCT05963399). The project team and participating organisations declared no conflicts of interest; all funding sources were fully disclosed in the manuscript. The research was conducted following strict governance and ethical standards for studies involving human subjects, ensuring confidentiality and data protection throughout |

1. M: Mandatory item
2. *Stages adapted from WHO Digital Health Atlas*
3. NM : Non-mandatory item
