# Supplementary material for: A Multidimensional Digital Health Platform to Support Intrinsic Capacity and Healthy Aging: Pilot Feasibility Study
Source: JMIR Aging. 2026 Jul 28;9:e79862. doi: 10.2196/79862 (PMC13462729; doi:10.2196/79862)
Supplement: Supplementary file 2 [file aging_v9i1e79862_app2.docx]

Multimedia Appendix n.1 Careup Positive Health application question select cycle. The questions selected on a given 4-week cycle day are marked with an ‘x’. The question identifiers match the labels in the CAREUP pilot dataset [59]
