# Supplementary material for: A Multidimensional Digital Health Platform to Support Intrinsic Capacity and Healthy Aging: Pilot Feasibility Study
Source: JMIR Aging. 2026 Jul 28;9:e79862. doi: 10.2196/79862 (PMC13462729; doi:10.2196/79862)
Supplement: Supplementary file 3 [file aging_v9i1e79862_app3.docx]

# Acceptance rating among primary users

| Questions |  | Respondents |
| --- | --- | --- |
|  |  |  |
| **1. How did you find the experience of using CAREUP?** | interesting, useful and efficient | 49 |
|  | too many devices with connection problems | 7 |
|  | over time, I expected more from the system | 5 |
| **2. What did you like about CAREUP?** |  |  |
|  | easy, useful, and new technology | 25 |
|  | health monitoring | 20 |
|  | games | 5 |
|  | smartwatch makes me feel active | 4 |
|  | mattress that assesses sleep quality | 3 |
|  | system slow, with too many devices, and not yet mature | 3 |
|  | no response | 1 |
| **3. What did you dislike about CAREUP?** |  |  |
|  | technical and connection problems bore and take time | 20 |
|  | nothing (I liked everything) | 27 |
|  | questionnaire questions are too repetitive | 5 |
|  | games | 5 |
|  | too many devices | 3 |
|  | questionable reliability of results | 1 |
| **4. Would you recommend CAREUP to a friend?** |  |  |
|  | yes | 48 |
|  | yes, if you have an interest in technology and health data collection | 6 |
|  | yes, if the system works and achieves long-term goals | 3 |
|  | I don't know | 2 |
|  | no | 2 |
| **5. Would you like to continue using CAREUP?** |  |  |
|  | yes | 39 |
|  | no | 11 |
|  | occasionally | 8 |
|  | yes, with fewer devices and periodically restocked | 2 |
|  | maybe | 1 |
| **6. Did CAREUP improve your ability to manage your daily routine?** |  |  |
|  | yes | 34 |
|  | no | 26 |
|  | I don't know | 1 |
| **7. Did CAREUP get in the way of your daily routine?** |  |  |
|  | no | 38 |
|  | yes | 21 |
|  | sometimes | 2 |
| **8. What tips would you provide to others who are new to the CAREUP system?** |  |  |
|  | use the system as much as possible | 17 |
|  | participate, it's easy and useful | 16 |
|  | have confidence and patience | 10 |
|  | familiarity with technology and reading a manual | 7 |
|  | ask for help, in case of need | 4 |
|  | no tips | 4 |
|  | follow instructions | 3 |
| **9. Do you have any other comments for us based on your experience using CAREUP?** |  |  |
|  | no | 17 |
|  | interesting games to be improved | 6 |
|  | improve technical and connection problems | 4 |
|  | a useful system for those who are sick, but it's too complex | 3 |
|  | training and manual required | 3 |
|  | reduce the number of devices | 3 |
|  | improve system interface, add physical exercises | 3 |
|  | no response | 22 |
| **10. Any suggestions to us?** |  |  |
|  | no | 24 |
|  | improve games | 6 |
|  | reduce the number of devices | 2 |
|  | extend the duration of the trial | 2 |
|  | improve instructions, maybe adding a video | 2 |
|  | improve system feedback and graph readability | 2 |
|  | add advice on diet | 1 |
|  | no response | 22 |

# Acceptance rating among caregivers

| Questions |  | Numerosity |
| --- | --- | --- |
|  |  |  |
| **1a. What was the best aspect of the CAREUP platform experience?** |  |  |
|  | continuous health monitoring | 8 |
|  | data storage, overview of health status | 3 |
|  | cognitive stimulation through games | 1 |
|  | good idea | 1 |
|  | undecided | 4 |
| **1b. And the worst?** |  |  |
|  | complex functioning of the system, to be improved | 5 |
|  | questionable management of health results | 2 |
|  | unclear graphs | 2 |
|  | continuous monitoring as a form of control | 1 |
|  | undecided | 7 |
| **2. What did you like about CAREUP?** |  |  |
|  | continuous health monitoring at home, data storage | 10 |
|  | cognitive games | 4 |
|  | a new idea with integrated app/devices | 4 |
|  | smartwatch makes you feel active | 2 |
|  | sense of caring | 1 |
| **3. What did you dislike about CAREUP?** |  |  |
|  | incorrect detections, technical problems, confusing interface | 9 |
|  | nothing (I liked everything) | 4 |
|  | trial too short | 2 |
|  | idea implementation | 1 |
|  | necessary familiarity with technology | 1 |
| **4. Would you recommend CAREUP to a person that is in charge of taking care of an older adult?** |  |  |
|  | yes | 8 |
|  | no | 4 |
|  | maybe | 4 |
|  | partially | 1 |
| **5. Would you like to continue using CAREUP?** |  |  |
|  | yes | 6 |
|  | maybe | 6 |
|  | no | 5 |
| **6. Did CAREUP get in the way of your daily routine?** |  |  |
|  | no | 10 |
|  | maybe | 4 |
|  | yes | 3 |
| **7. What tips would you provide to others who are new to the CAREUP system?** |  |  |
|  | try the system, be patient | 8 |
|  | first, take a look at the system and see if it interests you | 3 |
|  | no tips | 5 |
|  | don't forget to cultivate real relationships | 1 |
| **8. Do you have any other comments for us based on your experience using CAREUP?** |  |  |
|  | no | 14 |
|  | make the system simpler and improve technical problems | 3 |
